# An approach for aggregating upstream catchment information to support research and management of fluvial systems across large landscapes

**DOI:** 10.1186/2193-1801-3-589

**Published:** 2014-10-09

**Authors:** Yin-Phan Tsang, Daniel Wieferich, Kuolin Fung, Dana M Infante, Arthur R Cooper

**Affiliations:** Department of Fisheries and Wildlife, Michigan State University, 1405 S. Harrison Rd. Suite 115, East Lansing, MI 48823 USA; DiiKaTwenty9, East Lansing, MI USA; Department of Natural Resources, Institute for Fisheries Research, Ann Arbor, MI USA

**Keywords:** Digitized stream network, GIS, Aggregation, Landscape information, Large scale assessment

## Abstract

The growing quality and availability of spatial map layers (e.g., climate, geology, and land use) allow stream studies, which historically have occurred over small areas like a single watershed or stream reach, to increasingly explore questions from a landscape perspective. This large-scale perspective for fluvial studies depends on the ability to characterize influences on streams resulting from throughout entire upstream networks or catchments. While acquiring upstream information for a single reach is relatively straight-forward, this process becomes demanding when attempting to obtain summaries for all streams throughout a stream network and across large basins. Additionally, the complex nature of stream networks, including braided streams, adds to the challenge of accurately generating upstream summaries. This paper outlines an approach to solve these challenges by building a database and applying an algorithm to gather upstream landscape information for digitized stream networks. This approach avoids the need to directly use spatial data files in computation, and efficiently and accurately acquires various types of upstream summaries of landscape information across large regions using tabular processing. In particular, this approach is not limited to the use of any specific database software or programming language, and its flexibility allows it to be adapted to any digitized stream network as long as it meets a few minimum requirements. This efficient approach facilitates the growing demand of acquiring upstream summaries at large geographic scales and helps to support the use of landscape information in assisting management and decision-making across large regions.

**Electronic supplementary material:**

The online version of this article (doi:10.1186/2193-1801-3-589) contains supplementary material, which is available to authorized users.

## Introduction

Natural and anthropogenic landscape factors including climate and human land uses operate over large spatial extents to affect aquatic systems in a given location. Based in part on this understanding, freshwater ecologists incorporate a holistic view of freshwater systems that includes landscapes drained by waterbodies (Blanchet et al. 2009; Brown et al. 1996; Crosbie et al. 2012; Gudmundsson et al. 2012; Haddeland et al. 2011). This view is acknowledged as a "landscape approach," and numerous studies have shown how hydrologic, thermal, chemical, and biological properties of freshwater systems are influenced by landscape characteristics of their catchments (Allan 2004). Hydrologists and engineers also acknowledge the influence of catchment characteristics as shown by the prevalence of basin-scale initiatives focused on freshwater systems, with examples including storm water management efforts, floodplain delineation, and development of nonpoint source pollution control strategies (e.g., Sprague and Gronberg 2012). Similarly, natural resource managers charged with conserving and protecting freshwaters increasingly incorporate a landscape perspective into management activities, expanding a historically site-focused view to address basin- or regional-scale influences on freshwater habitats (Palmer et al. 2008; Poiani et al. 2000).

Accounting for landscape-scale influences on aquatic systems has been facilitated through data and approaches developed with Geographic Information Systems (GIS). With GIS, measured (i.e., by satellites, by census) or modelled estimates of various landscape information can be attributed within spatially-explicit units such as catchments of freshwater systems. For instance, high-resolution coverages of landscape features like vegetation and/or soil allow for understanding spatially-explicit controls on catchment hydrology. Future and current climate data may also be mapped or modelled to differentially characterize influences across catchments. Also, mapped locations of human land uses and anthropogenic disturbances allow managers and decision makers to evaluate and prioritize management actions across large regions to improve and protect aquatic habitats. Such work is being conducted by multiple local, state, and federal organizations and initiatives throughout the United States, with examples of federal agencies working over large extents including the U.S. Fish and Wildlife Service (e.g., Landscape Conservation Cooperatives http://www.fws.gov/landscape-conservation/lcc.html) and US Geological Survey (e.g., Aquatic GAP Program http://gapanalysis.usgs.gov/, Climate Science Centers http://www.doi.gov/csc/index.cfm).

**Figure 1:**
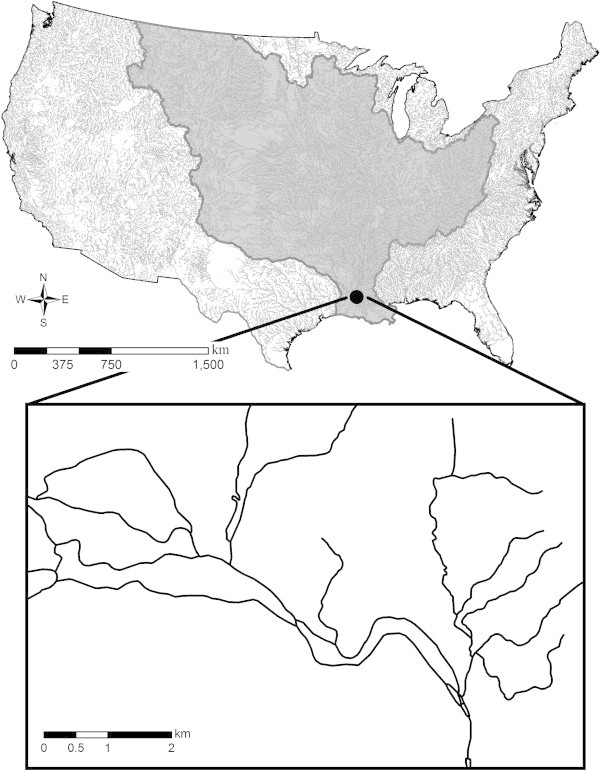
**The 1:100,000 NHDPlusV1 stream network over the conterminous U.S.** The Mississippi basin is emphasized in dark gray. The inserted box shows an example of complexity within stream networks, including an example of a braided stream.

Despite the importance of landscape-scale studies to management efforts for freshwater systems, such studies are challenged by the need for summarizing and synthesizing information over large areas. One contributing factor stems from the historical lack in consistency in describing discrete river reaches and their catchments over large areas. This challenge, however, is being addressed in part by development of extensive coverages of river networks (e.g. NHD, http://nhd.usgs.gov/), as well as by descriptions of spatial frameworks that incorporate standard definitions of rivers and catchments for analysis (i.e., Wang et al. 2011, Sowa and Annis 2007). Another contributing factor is the dendritic nature of river networks. Morphologically analogous to a tree, river systems accumulate water and substances from upstream tributaries and their respective subcatchments, yet the dendritic form of rivers network may lead to difficulties in summarizing and accounting for these influences. Examples of such upstream influences include numbers of point source pollutant sites occurring along a river network as well as nonpoint source pollutants (e.g., excess nutrients) drained from agricultural lands within river catchments. With GIS, such landscape information can be represented as point locations along river networks, or polygons or grid coverages over catchments. While the upstream information for one given stream location can be attributed and summarized easily, iteratively generating upstream summaries of such information for every stream location in a network throughout a large region can result in processing challenges, which can render the process unwieldy, exceeding typically-accessible computer processing capabilities. A further complication exists for braided river channels. Braided channels often occur near river mouths of large river basins, where stream power may increase, width to depth ratios may increase, and/or the amount and type of bedload may increase. In braided streams, stream channels become divided by multiple small bars or islands, and upstream summary of information requires explicit characterization of all upstream fluvial pathways. When river networks incorporate waterways that are braided, accounting for multiple pathways complicates the upstream summarization process, leading to various inaccuracies in aggregation of upstream information (insert within Figure 1). These challenges are exacerbated when landscape-scale studies for freshwater systems attempt to incorporate multiple landscape information layers. Summarizing information from multiple layers for every stream throughout a large region becomes a tremendous workload requiring substantial processing time.

To address the challenges of summarizing landscape information within river systems throughout large regions and to accurately summarize the information throughout braided river networks, we developed an approach to acquire summaries of upstream landscape information for every stream in a river network, including networks with braided channels. In applying this approach, we have confirmed accurate and consistent summaries of information over very large regions, including the conterminous Untied States. This approach can be applied to any river coverage with network topology defined and can include summary of landscape information from within catchments or from the river network itself. This paper presents detailed information on this approach and offers suggestions for applying it to river networks of interest.

### Requirements for the stream network layer

**Figure 2:**
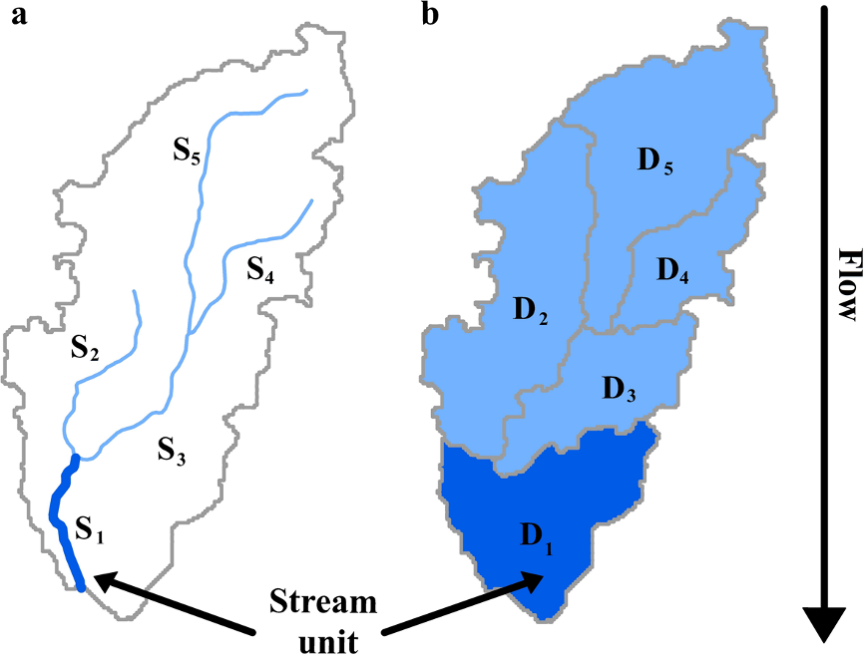
**An illustration shows the stream units in two types of digital stream networks. (a)** a polyline example, **(b)** a polygon example. This also illustrates that upstream landscape summary of S_1_ is a combination of local information at unit S_1_, and upstream information at units S_2_, S_3_, S_4_, and S_5_. Similar statement is applied to **(b)** with a polygon example.

Three requirements are necessary to apply our approach to acquire upstream summaries of landscape information from throughout river networks and their catchments. First, the stream network must be available in a digital geospatial format, referred to here as a digital stream networks. For our approach, digital stream networks can be represented in one of two types of vector mapping layers (Figure 2). One type includes a polyline layer that delineates the stream network including headwaters, tributaries, mainstems, and line junctions characterising points at which these fluvial bodies intersect. An example is the National Hydrography Dataset (NHD, http://nhd.usgs.gov/) for the United States. The second option is a polygon layer representing areas within a stream network that drain to specific sections of the streams. An example is the layer of functional elementary catchments (FECs) within European catchments and Rivers network system (Ecrins) ( http://www.eea.europa.eu/data-and-maps/data/european-catchments-and-rivers-network). Some digital stream networks include both polyline and polygon layers, and one example is the National Hydrography Dataset Plus Version 1 (NHDPlusV1, http://www.horizon-systems.com/NHDPlus/NHDPlusV1_home.php) for the conterminous United States. These digital stream networks provide a spatial framework in linking geospatial and landscape information (e.g. climate, soil, landuse) to catchments and reaches of the represented river network. The second requirement for our approach is that these polylines or polygons are broken into discrete units, such as stream segments or drainage areas of digital stream networks (Figure 2). Each unit in the network must be assigned a unique identifier. These unique and discrete units are referred to as stream units hereafter, and spatial information can be attributed and associated to these units. Finally, the third requirement is a key piece of information that describes network topology. Each stream unit needs an attribute that indicates the identifiers of the immediately upstream units. Figure 2a shows two stream units S_2_ and S_3_ that are the immediate upstream of the stream unit S_1_, and Figure 2b shows two stream units D_2_ and D_3_ that are the immediate upstream of stream unit D_1_. Both of the upstream units (i.e. S_2_ and S_3_, or D_2_ and D_3_) need to be indicated as occurring above unit S_1_ or D_1_, respectively. Identifying upstream units of a given stream unit requires knowledge of the flow direction within a stream network, and such information can be generated using elevation maps and GIS processing steps if it is not already incorporated into an existing digital stream network dataset. This information on network topology is essential for describing where a stream unit is located in a network. It stores the spatial relationship among stream units, ultimately allowing us to rebuild the context of the stream network to incorporate into the process of acquiring upstream summaries.

## Challenges of aggregating information throughout river networks

Summarizing landscape information for individual stream units from entire upstream networks, referred to following as "aggregation," has two unique challenges associated with dendritic fluvial networks: 1) the need to aggregate information over large spatial extents for every stream unit and 2) the need to account for braided streams.

### Large spatial extents

Studying streams using a landscape approach, in many cases, means evaluating stream networks comprising large systems (i.e., Mississippi River basin, Figure 1) or studying many stream systems within a large region (i.e., all streams in a state). As previously stated, when characterising a stream unit, influences originating from all upstream units need to be considered. A common approach includes delineating upstream networks for a stream unit and attributing landscape information to the unit using GIS. Similar processes are then repeated for all units of interest. Programming the process and computing within GIS, in particular, requires using large, cumbersome spatial files for information summary and as well as large amounts of storage space and computational capacity. The time and resources required for processing these spatial files of large region often overwhelm the memory and computational capacity of a standard computer and GIS software. These issues are further complicated if multiple layers of landscape information need to be aggregated over a large region.

Although existing tools have been developed to generate upstream landscape summaries for all stream units within a given stream network, they often have limitations that hamper their usefulness when applied across large geographic regions, or they may be built for specific datasets limiting their transferability. One example, the Catchment Attribute Allocation and Accumulation Tool (CA3T), developed by Horizon System Corporation ( 2008), provides a process for aggregating upstream information for stream units of the NHDPlusV1. When producing upstream summaries for units of interest, the tool draws from NHDPlusV1 Tools Application Data, a large set of application files (about 1 GB total size) that indicate stream network topology. This information, along with tabular data of each stream unit, forms the basis of the aggregation process when using this tool. Because the stream networks of the conterminous U.S. are divided into 18 regions within the NHDPlusV1, it is important to know a priori which regions are located upstream of the target region, requiring the user to append upstream tabular data in CA3T in order to correctly generate upstream summaries. In addition to the NHDPlusV1 Tools Application Data, the software requirements for CA3T include either ArcGIS 9.2 or 9.3, including the ArcGIS Spatial Analyst extension and service packs, and .Net Framework version 2.0 in order to run the attribution and aggregation process. This combination of files and software applications (and their interaction) can demand intensive processing time within the larger regions of the NHDPlusV1. Further complications include the development of newer versions of ArcGIS and .Net Framework software since the development of CA3T, requiring the user to identify and use the correct versions of these respective software. A second example of an existing tool that generates upstream landscape summaries includes the Arc Hydro tool. Arc Hydro is a comprehensive tool that is regularly updated and supported with release of new versions of ArcGIS. It can perform terrain processing (e.g. digital elevation model (DEM) manipulation and flow direction) as well as watershed processing (e.g. watershed delineation). It also has functions that attribute and aggregate landscape data from throughout river networks. However, it is important to note that standard application of the aggregation function must be performed on files generated from previous sequences of steps in the Arc Hydro process (i.e. terrain processing and watershed processing). In other words, to perform aggregation, users need to start from the DEM manipulation (including creation of catchment boundaries) in order to have files from previous steps. For cases with predefined digitized stream networks and existing catchments like NHDPlusV1, it would take a significant amount of effort to adapt the aggregation function in Arc Hydro. In particular, when it comes to aggregation for large region, Arc Hydro along with other general extensions developed for ArcGIS, such as Network Analyst, do not have the capacity to provide upstream aggregations due to memory limitations. The constraint of current aggregation options emphasizes the need of an approach that is convenient, flexible, and efficient for large-scale aggregation.

### Braided streams

**Figure 3:**
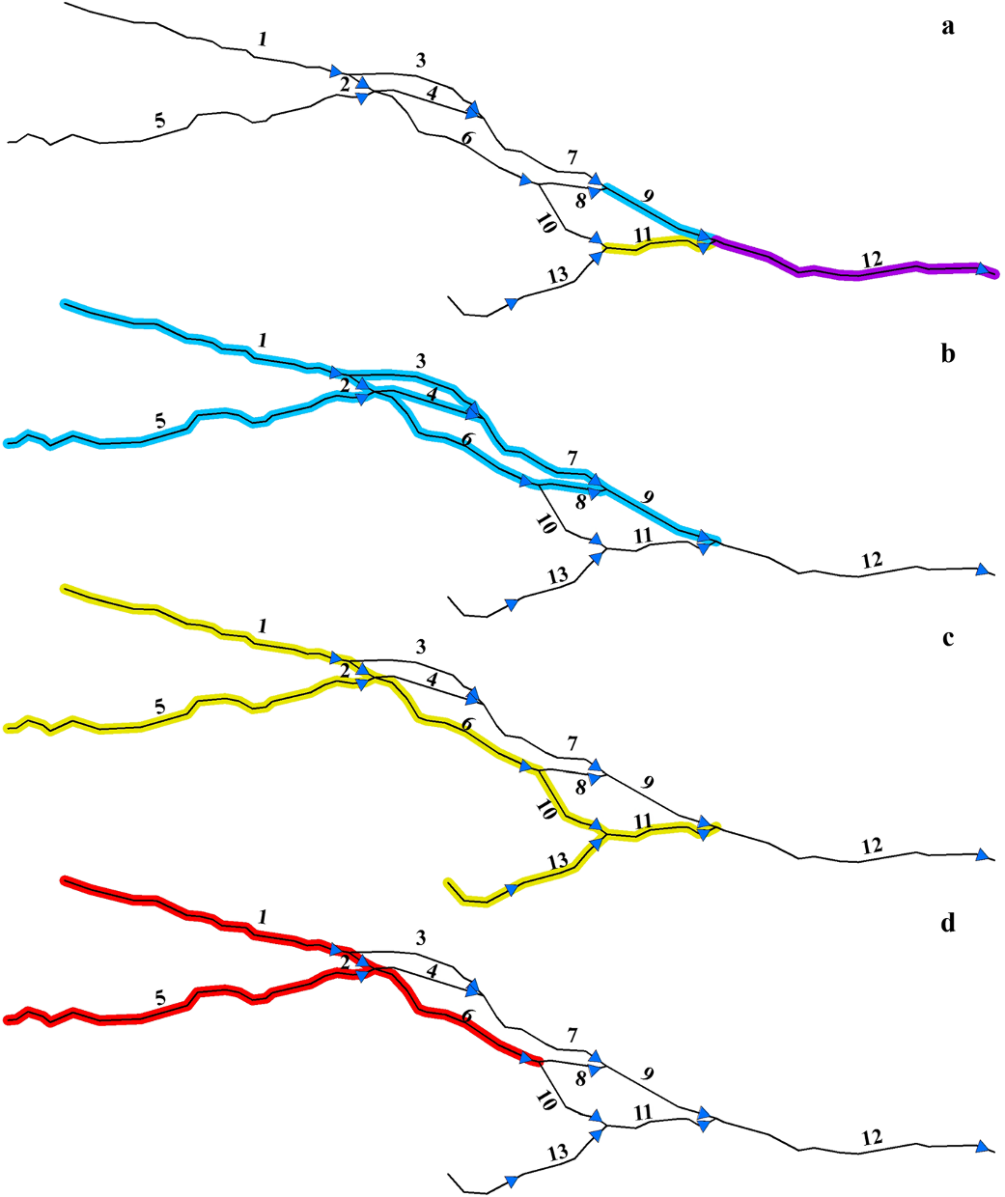
**A hypothetical example of braided streams. a**. the immediate upstream units of unit 12, **b**. the upstream network of unit 9, **c**. the upstream network of unit 11, **d**. the overlap of the stream network of units 9 and 11.

Braided streams are common in fluvial networks, especially near mouths of large river basins. For example, see Figure 1 depicting braids near the mouth of the Mississippi River, or see Figure 3 for a more complex break down of a braided river network. In Figure 3, the upstream network of unit 12 includes units 9 and 11 along with all units upstream of these two units. (Figure 3-a). The upstream network of unit 9 includes units 1, 2, 3, 4, 5, 6, 7, 8 (Figure 3-b), while the upstream network of unit 11 includes units 1, 2, 5, 6, 10, 13 (Figure 3-c). Units 9 and 11 have overlap in a set of units including 1, 2, 5, 6 (Figure 3-d). This reflects the challenge of acquiring upstream summaries for unit 12; the aggregation process must ensure summarization does not duplicate information of shared units in the upstream network. Moreover, the braided network represented in Figure 3 is a fairly simple network structure. In many cases, braided systems may be much more complex, with a stream located within such networks having multiple immediate upstream and downstream units, further complicating the aggregation process and underscoring the need for an algorithm that describes the context of braided streams while allowing for summary of information in a non-duplicative manner for a stream unit of interest.

## The aggregation approach

**Figure 4:**
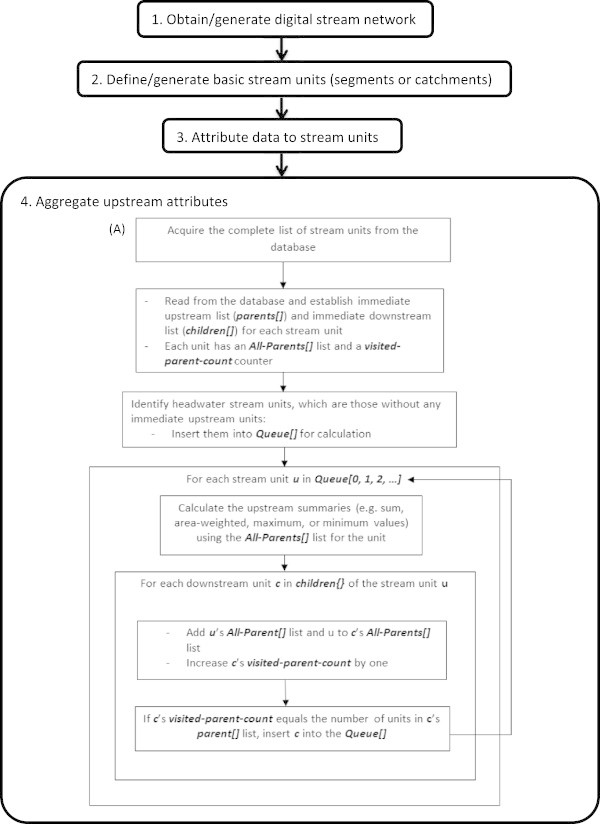
**The flow chart of performing upstream aggregation. (A)** the developed algorithm to build stream network context.

Our approach consists of two main components: 1. building a database that meets requirements for performing aggregation, and 2. applying an algorithm that interacts with the database to aggregate information for all stream units throughout large regions and correctly for braided networks. Figure 4 shows the complete steps to perform our aggregation approach.

### Building a database for performing aggregation

All discrete units of the digitized stream network must be referenced in the database with unique identifiers which are used as primary keys in the database. These units along with the immediate upstream units of each unit are the foundation of the database necessary for aggregating information. Also within the database, landscape information of interest for aggregation should be incorporated for each unit. Many types of information may be attributed for stream units within river networks. Examples include numbers of barriers or road crossings located on a stream segment, or water quality data such as numbers of point source discharges located on streams. Often, however, attribution of landscape coverages within catchments is a focus of the aggregation process. Examples of coverages that may be useful for research or modeling efforts include summaries of amounts of forested land cover, agricultural land use, or impervious surface within catchments. Such landscape information is often initially available as continuous grid data for regions of interest, and before aggregation, must be attributed to stream units. Attributions of landscape information can be incorporated into the database as records specific to each unit. Attributing information can be accomplished in various ways depending on data type. For example, the ArcGIS Spatial Analyst extension Tabulate Area and Zonal Statistics functions can be used for grids or polygon data, which are often how land cover data are represented. When catchment summaries of landscape information are of interest, we also recommend including catchment areas for each unit as an additional attribute in the database because landscape information can sometimes require summary by area-based weighting. Database development and management may occur with a variety of software, including open source (e.g. MySQL, Firebird) or commercial software (e.g. Oracle, Microsoft SQL Server).

### Applying an algorithm for aggregation

Database requirements described above allow for re-creation of the context of the river network by applying our developed algorithm script, which reads and writes information off of this database. As this is a tabular process (vs. one requiring summary of information directly from spatial data in a GIS environment), aggregation with our algorithm can occur quickly throughout very large regions. Further, multiple types of landscape information may be summarized simultaneously.

Our algorithm was written in Ruby ( http://www.ruby-lang.org/en/), and a flow chart of the algorithm is shown in Step 4 of Figure 4. We developed the algorithm to recreate the stream network context from headwater streams to the most downstream reach in a given network. At the beginning of the process, the program acquires a complete list of unique identifiers for stream units in the network. For each unit, a list of immediate upstream units, *parents[]*, and a list of immediate downstream units, *children[]*, are established using the attribute from the database identifying the immediate upstream units. Additionally, an *All-parents[]* list, which begins as an empty list, is established to keep track of all the identifiers of units within the upstream network for each individual unit. The algorithm first identifies headwater units, because headwaters have no immediate upstream units in their *parents[]* list. The algorithm then adds each of these headwater units into the queue list, *Queue*[] for calculating aggregation summaries. Remaining units include those units with immediate upstream units in their *parents[]* list. In many cases, the *parents[]* list for a given unit contains only one immediate upstream unit. In the cases of confluences, the *parents[]* list of downstream unit contains two immediate upstream units, and the two upstream units have the same downstream unit in their c*hildren[]* lists. The algorithm adds these two upstream units along with their *All-parents[]* lists to the *All-parents[]* list of the downstream unit. Further, each stream unit has a counter, *visited-parent-count*. When the *visited-parent-count* equals the number of units in the *parent[]* list (which means all upstream units of the downstream unit are included), the unit is added to the *Queue[]*, for performing the upstream summary.

Our algorithm also performs aggregation of information throughout braided streams effectively. This is due to the fact that when a list is built, it is established as a "set," a data structure in Ruby which implements a collection of unordered values with no duplicates. Therefore, this eliminates the problem of double-counting upstream information for braided streams in the aggregation process.

Our algorithm can be used to perform various calculations, including searching for maximum or minimum values of summaries in the list (which will reflect maximum or minimum values of spatial information on stream or over the catchment within stream networks). Also, the sum of information from within stream network can be calculated, reflecting the total count of certain characteristics within stream networks. As a final example, area-weighted catchment summaries of landscape information may be calculated from across all upstream subcatchments for describing patterns in the basin. While these are examples of commonly applied summaries, many additional calculations could be conducted to summarize information from throughout the stream network. These various types of calculations can be incorporated into the algorithm with results output to a single database, minimizing the time needed to organize input and output data. (Note: This program "UpstreamAggregationExampleCode.rb" is available as Additional file 1 for readers’ reference).

## Evaluation of our approach

We evaluated our aggregation approach in three ways. First, we used our approach to summarize urban and agricultural land uses (National Land Cover Database 2001, Homer et al. 2007) for catchments of 2.3 million stream units within the conterminous United States as represented by the NHDPlusV1 (Figure 1). We compared these aggregated summaries with the same summaries achieved using the CA3T tool. Results were comparable, supporting the accuracy of our aggregations. Next, results for about two hundred stream units were manually verified. Manual inspections were focused on areas of the network with braided streams, yet various positions within the stream network were verified to ensure accuracy. Finally, we evaluated the maximum number of landscape variables that could be aggregated at once without a substantial reduction in processing time using our tool. We found that we could aggregate up to 24 landscape variables for all stream reaches of the conterminous United States in 5 hours (with XEON QUAD CORE E5620 processor and 12G RAM, and using MySQL database software) with no substantial change in processing time. This efficiency is expected given that the aggregation process uses a database as opposed to using spatial data files. In particular, this program performs aggregation of all streams within the program and accesses database only at the beginning and at the end of the process, which limits the time spent in database input/output. This approach allows acquisition of landscape summaries in a timely manner and facilitates stream research and management efforts at a landscape scale.

## Conclusion and discussion

This approach was developed due to the need for efficiently aggregating landscape information throughout catchments of all streams in the conterminous United States. It builds the stream network context from headwater streams to downstream units, and aggregates summaries of information throughout the basins. In particular, it accurately acquires summaries through braided streams without double counting of values on streams and their catchments. This approach needs neither GIS spatial files nor additional software applications in the process; therefore it will not be obsolete due to software updates. This approach requires building a database and applying a programming algorithm, yet it is not confined by any particular database software or specific programming language. Despite the original purpose of large regional aggregation, this approach could be used for summarizing information in smaller regions, and it could be applied to any geographical area as long as stream units comprising a network are identified with unique identifiers and have associated topology. We have applied this approach to different areas with digitized stream networks (e.g., 1:24,000 NHD in Hawaii; http://nhd.usgs.gov/), and as new stream layers become available with the necessary criteria (e.g., NHDPlusV2, http://www.horizon-systems.com/NHDPlus/NHDPlusV2_home.php), one can aggregate information to the new digitized stream network, ensuring that this approach will be useful into the future.

With the increasing availability and quality of images and surveys, GIS has been widely adopted and applied to many fields, including freshwater ecology, hydrology, and engineering with efforts directed at understanding and managing river systems. The described aggregation approach will promote the use of geospatial data in these disciplines by providing summaries of upstream information for stream networks. Management of water resources could use these summaries to inform decision making about freshwater resources. An example application is using aggregated information from upstream networks to enhance understanding of controls on or limits to stream reaches. Historically, stream management and restoration efforts have been criticized when they adopt a narrow focus vs. considering watershed influences (Palmer et al. 2010). Because streams are closely connected with other ecosystems, such as terrestrial, estuary, and coastal ecosystems, studies and management of these ecosystems could also benefit from information of upstream summaries in planning conservation management of their ecosystems of interest.

## Electronic supplementary material

Additional file 1: **The upstream aggregation algorithm written in Ruby.** (UpstreamAggregationCode.rb). (ZIP 4 KB)
